# A Cross-Sectional Study of the Street Foods Purchased by Customers in Urban Areas of Central Asia

**DOI:** 10.3390/nu13103651

**Published:** 2021-10-19

**Authors:** Sofia Sousa, Inês Lança de Morais, Gabriela Albuquerque, Marcello Gelormini, Susana Casal, Olívia Pinho, Carla Motta, Albertino Damasceno, Pedro Moreira, João Breda, Nuno Lunet, Patrícia Padrão

**Affiliations:** 1 EPIUnit-Instituto de Saúde Pública, Universidade do Porto, Rua das Taipas 135, 4050-600 Porto, Portugal; sofia.sousa@ispup.up.pt (S.S.); gabriela.albuquerque@ispup.up.pt (G.A.); sucasal@ff.up.pt (S.C.); tino_7117@hotmail.com (A.D.); pedromoreira@fcna.up.pt (P.M.); nlunet@med.up.pt (N.L.); 2 Laboratório para a Investigação Integrativa e Translacional em Saúde Populacional (ITR), Rua das Taipas 135, 4050-600 Porto, Portugal; 3 Faculdade de Ciências da Nutrição e Alimentação da Universidade do Porto, Rua do Campo Alegre 823, 4150-180 Porto, Portugal; oliviapinho@fcna.up.pt; 4 Nutrition, Physical Activity and Obesity Programme, Division of Noncommunicable Diseases and Life-Course, World Health Organization (WHO) Regional Office for Europe, UN City, Marmorvej 51, 2100 Copenhagen, Denmark; inesbolm@gmail.com (I.L.d.M.); marcello.gelormini@gmail.com (M.G.); 5 REQUIMTE, Laboratório de Bromatologia e Hidrologia, Faculdade de Farmácia, Universidade do Porto, Rua D. Manuel II, Apartado, 4050-313 Porto, Portugal; 6 Departamento de Alimentação e Nutrição, Instituto Nacional de Saúde Doutor Ricardo Jorge (INSA), Avenida Padre Cruz, 1649-016 Lisboa, Portugal; carla.motta@insa.min-saude.pt; 7 Departamento de Ciências da Saúde Pública e Forenses e Educação Médica, Faculdade de Medicina da Universidade do Porto, Alameda Prof. Hernâni Monteiro, 4200-319 Porto, Portugal; 8 Faculdade de Medicina, Universidade Eduardo Mondlane, Avenida Salvador Allende 702, 257 Maputo, Mozambique; 9 Centro de Investigação em Atividade Física, Saúde e Lazer, Universidade do Porto, Rua Dr. Plácido Costa 91, 4200-450 Porto, Portugal; 10 WHO Regional Office for Europe, UN City, Marmorvej 51, 2100 Copenhagen, Denmark; rodriguesdasilvabred@who.int

**Keywords:** street food, nutritional value, nutrition transition, central Asia

## Abstract

This study aimed to describe street food purchases in cities from Central Asia, considering customers’ characteristics and the nutritional composition of the foods and beverages. Cross-sectional studies were conducted in 2016/2017 in Dushanbe (Tajikistan), Bishkek (Kyrgyzstan), Ashgabat (Turkmenistan) and Almaty (Kazakhstan). Direct observation was used to collect data on the purchases made by street food customers, selected by random and systematic sampling. Nutritional composition was estimated using data from chemical analyses, food composition tables or food labels. A total of 714 customers (56.6% females, 55.5% aged ≥35 years, 23.3% overweight/obese) were observed, who bought 852 foods and beverages, the most frequent being savoury pastries/snacks (23.2%), main dishes (19.0%), sweet pastries/confectionery (17.9%), tea/coffee (11.3%) and soft drinks/juices (9.8%). Fruit was the least purchased food (1.1%). Nearly one-third of customers purchased industrial food items (31.9%). The median energy content of a street food purchase was 529 kcal/serving. Saturated and *trans*-fat median contents were 4.7 g/serving and 0.36 g/serving (21.4% and 16.5% of maximum daily intake recommendations, respectively). Median sodium and potassium contents were 745 mg/serving (37.3% of maximum recommendation) and 304 mg/serving (8.7% of minimum recommendation), respectively. In general, the purchases observed presented high contents of energy, saturated-fat, *trans*-fat and sodium, and low levels of potassium. Policies towards the improvement of these urban food environments should be encouraged.

## 1. Introduction

Non-communicable diseases (NCD) are the first cause of mortality and disability in the world, and more than three-quarters of the NCD deaths (32 million) occur in low- and middle-income countries (LMIC) [1]. In Central Asian countries, NCD accounted for more than two-thirds of the deaths in 2016, mostly due to cardiovascular diseases and cancer [2]. The prevalence of obesity has been rising rapidly in this region, with a rate of increase between 2010 and 2016 ranging from 3.2% per year in Kazakhstan to 4.7% per year in Tajikistan. In 2016, obesity affected 12.6% of adults in Tajikistan, 15.4% in Kyrgyzstan, 17.5% in Turkmenistan and 21.3% in Kazakhstan [3]. High blood pressure affects one in every four adults in these populations [2].

The increasing prevalence of diet-related NCD in LMIC has been concurrent with the westernization of food habits and closely related to economic growth, rapid urbanization and globalization of the food supply [4,5]. In developing Asia, the consumption of soft drinks and high-fat processed foods has been increasing, and a growing proportion of dietary energy from fat and refined sugars has been reported [6]. Sodium intake is also high, corresponding to approximately three times the recommended amount [2]. These nutritional and epidemiological changes are expected to be even more pronounced in the most urbanized areas [7].

Street food is frequently consumed worldwide, with an estimated 2.5 billion people eating street food on a daily basis in 2007; the main reasons for consumption included low cost and convenience [8]. In LMIC, street food has a significant dietary contribution to the everyday diet, frequently serving as a replacement for home meals [9]. In Central Asia, there is a strong tradition of selling ready-to-eat foods on the streets, mostly in the context of organized public markets [10,11,12,13]. Although local traditional foods remain widely available, industrialized foods and ingredients are becoming more prevalent [8]. Recent studies from the World Health Organization (WHO) reported a wide availability of soft drinks, as well as concerning levels of sodium and *trans*-fat in both homemade and industrial street foods in cities from Central Asia [10,11,12,13].

Despite the importance of street foods in LMIC, scientific research covering its availability, consumption and nutritional value is scarce, and considered crucial to understand its implications for public health [14] and ultimately to design suitable policies towards diet-related NCD prevention. The FEEDCities project was created to bridge this gap of knowledge in urban areas from central Asian and eastern European LMIC, and have already provided valuable data regarding food availability, as well as the nutritional value of some highly available foods in these settings [10,11,12,13]. However, although food availability is an important feature of the food environments description, it does not directly reflect food choices. In addition, in this previously published work only data from chemical analyses of the most frequently available food items (approximately 30 foods and beverages out of 100 identified, with slight variations among cities) were reported. Thus, the assessment of street food customers and their purchases, including the nutritional composition of each purchase as a whole instead of individual food items, is essential to complement availability data, in order to have a much more comprehensive view of both environmental and individual dimensions of these street food environments. As such, the main objective of this study was to describe the street food purchases in urban areas of Tajikistan, Kyrgyzstan, Turkmenistan and Kazakhstan, taking into account customers’ characteristics and the nutritional composition of the foods and beverages purchased. In this study, we go beyond the nutritional characterization of the most available foods, by assessing what was purchased by customers, regardless of the frequency in which the food is available for sale.

## 2. Materials and Methods

**Figure 1:**
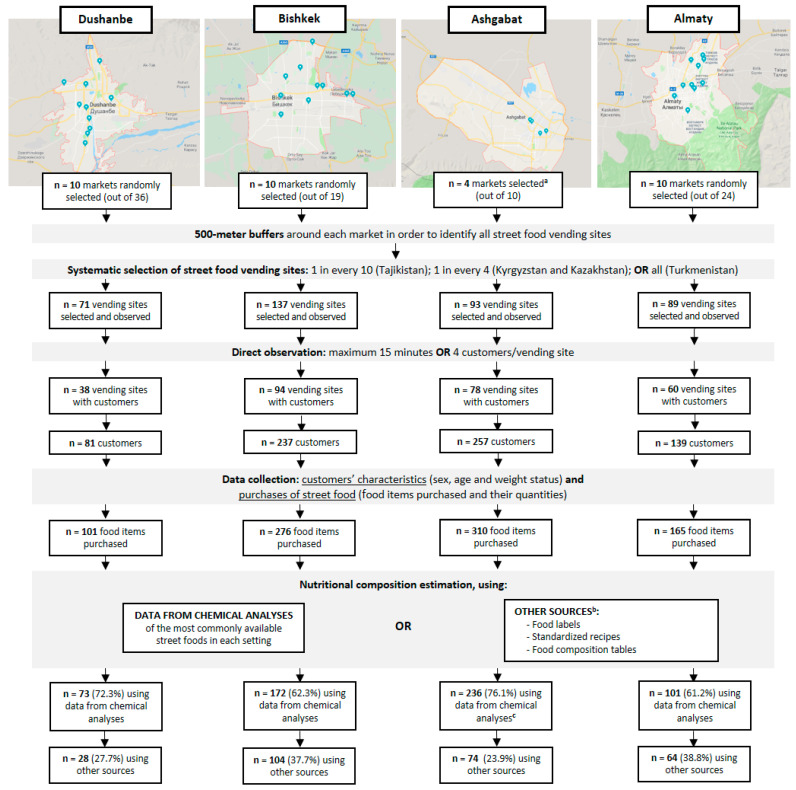
Flow chart of the sampling procedure and data collection conducted in *Dushanbe* (Tajikistan), *Bishkek* (Kyrgyzstan) *Ashgabat* (Turkmenistan) and *Almaty* (Kazakhstan). ^a^ In Turkmenistan, markets to be studied were selected by the public authorities in the country. ^b^ For the least commonly available street foods in each setting, or those whose nutritional composition was considered to be well documented (tea, coffee, soft drinks, water and fruit). ^c^ In Turkmenistan, exceptionally, the samples collected for chemical analysis were not analysed for protein and carbohydrates contents, those being estimated using food composition tables, standardized recipes or food labels.

This study was implemented in the context of the FEEDCities (Food Environment Description in cities from Central Asia and Eastern Europe) project, which is based on a stepwise standardized characterization of the street food environment in cities from Central Asia and Eastern Europe. For the purpose of this work, a cross-sectional evaluation of street food customers was conducted in *Dushanbe*, *Bishkek*, *Ashgabat* and *Almaty*, the largest and most urbanized cities of Tajikistan, Kyrgyzstan, Turkmenistan and Kazakhstan, respectively [15]. The surveys were conducted during April and May 2016 in *Dushanbe*, June and July 2016 in *Bishkek*, October 2016 in *Ashgabat*, and July and August 2017 in *Almaty*. The general methods were described in a previously published protocol [16] and detailed below. A flow chart summarizing the sampling procedures and data collection is presented in Figure 1.

### 2.1. Eligibility Criteria

The definition of street food proposed by the Food and Agriculture Organization (FAO) and the World Health Organization (WHO), as “ready-to-eat foods and beverages prepared and/or sold by vendors or hawkers especially in the streets and other similar places” [17,18], was adopted in order to select the street food vending sites where participants were selected. This includes both stationary and mobile vending units selling, directly to the street, food products ready to be consumed immediately without needing further preparation, comprising prepared (e.g., sandwiches), cooked (e.g., main dishes), *in natura* (e.g., fruits) or industrial foods (e.g., soft drinks, candies).

The exclusion criteria were as follows: (1) food establishments within four permanent walls or permanent storefront business not selling directly to the street; (2) vending sites selling exclusively non-prepared fresh fruits and vegetables or other raw foods not ready-to-eat (e.g., fish, meat); (3) food stalls and carts that are part of permanent stores or licensed establishments.

Customers buying ready-to-eat foods and/or beverages in the selected street food vending sites during pre-specified periods were eligible for the study.

### 2.2. Sampling Procedure

All public markets in *Dushanbe* (*n* = 36), *Bishkek* (*n* = 19), *Ashgabat* (*n* = 10) and *Almaty* (*n* = 24) were identified, with information provided by local authorities and gathered during preliminary field visits, as previously described [10,11,12,13,19,20]. In each city, a total of 10 markets were randomly selected (in *Ashgabat*, exceptionally, four markets were chosen by local authorities). Around the centroid of each selected market, a 500-m buffer was defined to represent the study area, covering the markets and their surroundings. Field researchers, operating in pairs, canvassed all publicly accessible streets within the study areas of each city, identifying all eligible street food vending sites. Finally, a systematic selection of the vending sites was performed, based on the number of vending sites encountered in each study area and the expected overall number of customers, in which one out of every 10 (Tajikistan), one out of every four (Kyrgyzstan and Kazakhstan) and all vending sites (Turkmenistan) identified in each city were observed.

In each selected vending site, all customers buying any food or beverage which meets the abovementioned definition of street food were observed. The observation period started at the next multiple of five minutes and ended after 15 minutes or when four customers were observed, whichever came first. If no customer was observed during this period, field researchers would move on to the next selected vending site. Observations were performed both on weekdays and weekends and covering all businesses’ working hours (from 8 a.m. to 5 p.m.).

### 2.3. Data Collection

Data on customers’ characterization and street food purchase were collected by direct observation of each selected street food vending site during its regular activity, by local field researchers, who were located at a distance sufficient for not interfering with the vendors’ businesses or the normal behaviour of the customers. The body mass index (BMI)-based body size guides for women and men by Harris et al. [21] were used for training of the observers, aiming to improve and standardize the anthropometric evaluation by observation.

For each customer buying street food, two observers independently described the foods and beverages purchased (hereafter referred to as food items) and their quantities, as well as the customers’ sex and estimated age and BMI-based weight status, in broad categories. Inter-observer concordance was high to very high, regarding both the customers’ characteristics and the street food items purchased, as well as its quantities (Supplementary Table S1). For age and weight status, only data corresponding to the customers in which there was agreement between observers [*n* = 642 (89.9%) and *n* = 656 (91.9%), respectively] were used for data analysis. Regarding the food items and quantities purchased, a set of criteria was used in order to eliminate observation conflicts (Supplementary Table S2).

The purchased food items were classified according to their nature as: *homemade* (foods and beverages that were prepared and/or cooked at home or in the street, even if using industrial ingredients) or *industrial* (foods and beverages that were produced by the food industry and sold as such, with no further preparation). Ready-to-eat in natura foods (e.g., fruits, nuts) were also classified as homemade. Both foods and beverages were further grouped into six sub-categories each, considering similarities of nature or composition, based on the WHO nutrient profile model [22], namely: (1) main dishes, (2) breads, (3) savoury pastries and snacks, (4) sweet pastries and confectionery, (5) sandwiches, and (6) fruit. Beverages were divided into: (1) tea and coffee, (2) soft drinks and juices, (3) non-alcoholic fermented traditional beverages, (4) alcoholic beverages, (5) milk, and (6) water. For example, rice- and noodles-based dishes (e.g., *plov*, *lagman*) were grouped into the “main dishes” group and were also classified as “homemade”, since they were all cooked by vendors at home or in the street.

### 2.4. Nutritional Composition Estimation

The nutritional composition of all food items purchased was estimated using data either from chemical analyses (for the most commonly available food items in each setting) or from food labels, standardized recipes or food composition tables. The nutritional parameters estimated comprised energy, protein, carbohydrates, total fat, saturated fatty acids (SFA), monounsaturated fatty acids (MUFA), polyunsaturated fatty acids (PUFA), *n*−6 and *n*−3 fatty acids, trans fatty acids (TFA), sodium and potassium.

During the first step of the research project, which assessed street food availability, the most commonly available street foods and beverages in each setting were documented [10,11,12,13,19,20]. The 20 (*Bishkek* and *Almaty*), 21 (*Ashgabat*) or 25 (*Dushanbe*) most common homemade foods and the 10 (*Bishkek*, *Almaty* and *Dushanbe*) or 11 (*Ashgabat*) most common industrial foods were selected for chemical analysis, excluding tea, coffee, soft drinks, water and fruit, as their nutritional composition was considered to be well documented. Then, four samples of each food were collected in different randomly selected vending sites in each city. Chemical analyses, including macronutrients, fatty acids, sodium and potassium, were performed for all collected samples, and energy values were calculated using the Atwater general factors, all in accordance with the Association of The Official Analytical Chemists recommendations [23], as described elsewhere [10,11,12,13,19,20]. In *Ashgabat*, exceptionally, the samples collected were not analysed for protein and carbohydrates contents, those being estimated using food composition tables, standardized recipes or food labels, as described in detail below.

For the food items which were not sampled for chemical analysis, nutritional composition was estimated using food labels, standardized recipes or food composition tables. For industrial food items, nutritional information was gathered from food labels of the brands most commonly available in each setting. For prepared and/or cooked homemade food items, standardized local recipes were gathered in each country. In the case of in natura food items (e.g., fruits, nuts) as well as for recipe ingredients, nutritional composition was obtained from the Food Composition Table for Pakistan [24] or from the Turkish National Food Composition Database [25], selected due to geographical and cultural proximity. Information from food labels and food composition tables did not include fatty acids and, in most cases (80.8% and 98.2%, respectively), sodium and potassium contents. As such, only energy, protein, carbohydrates and total fat were estimated for all food items which were not subjected to chemical analysis. Whereas sodium content was estimated for industrial and in natura food items using food labels and food composition tables, in the case of prepared/cooked homemade foods and beverages, it was not estimated using these sources, since the amount of salt added during their preparation and/or cooking was considered to vary greatly, being exclusively estimated by chemical analysis.

The nutritional value of the street food purchases of each customer was then computed by adding up the estimated energy, macro- and micro-nutrients of all foods and beverages purchased by the same customer on a single occasion. Purchases not having the complete information of all food items regarding one specific nutrient, were considered as missing values for that nutrient.

WHO daily population nutrient intake goals were then computed: SFA, <10% of total energy value (TEV) [26]; TFA, <1% TEV [27]; sodium, <2000 mg [28]; potassium, ≥3510 mg [29]; and sodium to potassium molar ratio, 1 [28,29]. In order to estimate the nutritional contribution in relation to the maximum daily intake recommendations, a daily reference intake of 2000 kcal was considered.

### 2.5. Statistical Analysis

Statistical analysis was performed using *Stata*^®^ version 15.0 for *Windows*^®^. Absolute and relative frequencies were used to characterize street food customers and the food items purchased. The nutritional composition of the purchases was described through median and percentiles 25 and 75. Inter-observer concordance for the demographic and anthropometric characteristics of the customers, as well as for the food items purchased and their quantities, were analyzed through the percentage of agreement and Cohen’s kappa coefficient with 95% confidence interval. Pearson’s Chi-squared test, Mann–Whitney’s U test and Kruskal–Wallis test were used to compare the frequencies of the food items and the nutritional composition of the purchases regarding demographic and anthropometric characteristics of the customers observed. Differences were considered statistically significant when the critical level of significance (*p*) was less than 0.05.

## 3. Results

A total of 714 customers were identified, 81 in Tajikistan, 237 in Kyrgyzstan, 257 in Turkmenistan and 139 in Kazakhstan. Slightly more than half were female (56.6%) and aged 35 years and older (55.5%), and almost one-quarter (23.3%) were classified as overweight or obese. A total of 852 food items were purchased, corresponding to an average of 1.2 food items per customer and 3.8 food items per 10 minutes of observation. Most customers (83.3%) purchased only one food item, and more than two-thirds (69.1%) purchased only foods. Most customers purchased only homemade food items (68.1%), although nearly one-third purchased at least one industrial food or beverage (31.9%); this proportion was highest in Kazakhstan (43.2%) (Table 1).

**Figure 2:**
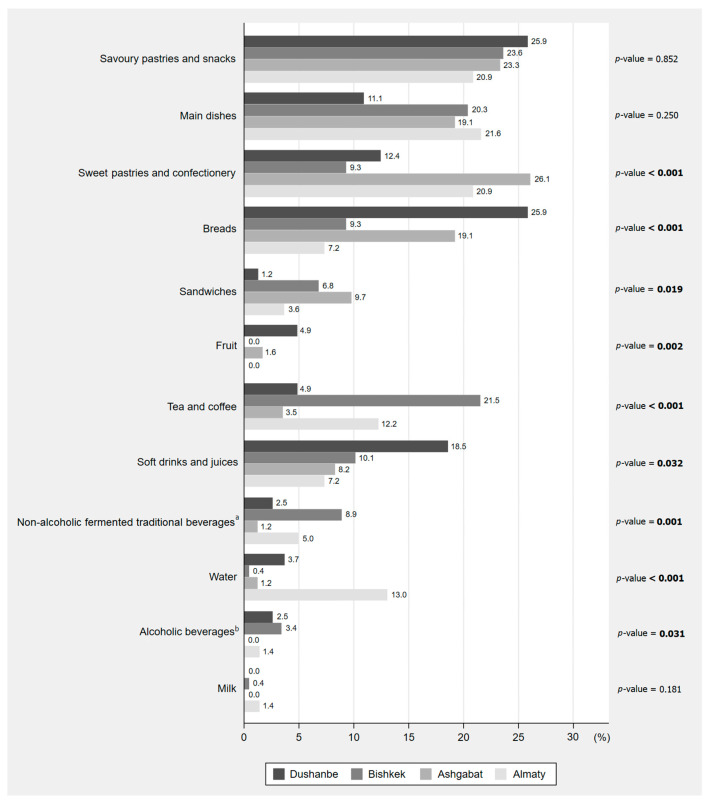
Ready-to-eat foods and beverages purchased by the customers observed in street food vending sites of Dushanbe (Tajikistan), Bishkek (Kyrgyzstan) Ashgabat (Turkmenistan) and Almaty (Kazakhstan) (*n* = 714). The proportions presented are relative to the total number of customers observed in each country (Tajikistan, *n* = 81; Kyrgyzstan, *n* = 237; Turkmenistan, *n* = 257; Kazakhstan, *n* = 139). The sum of the percentages from each country may exceed 100% because the same customer could buy more than one food item. Values in bold represent statistically significant differences according to Pearson’s Chi-squared test with a significance level of 0.05. ^a^ Non-alcoholic fermented traditional beverages included *ayran* (dairy-based fermented beverage made from sheep’s milk), *chalap* (beverage made from fermented milk, salt and carbonated water;), *dugob* (fermented beverage made with sour milk or buttermilk), *kefir* (fermented milk drink prepared by inoculating cow, goat or sheep milk with kefir grains), *maksym* (fermented beverage made from grain, usually malt), *tamshan* (mix of *maksym* and *chalap*), *kozhe* (cold drink made by boiling rice, millet or pearl barley with a mixture of dairy products such as *ayran* or *kefir*) and yoghurt. ^b^ Alcoholic beverages included beer, vodka and some traditional beverages with a low alcohol content, such as *kvass* (a fermented beverage made from rye bread), *bozo* (a fermented beverage made from millet) and *kymyz* (a fermented product made from mare’s milk).

Overall, the most commonly purchased foods were savoury pastries and snacks (23.2% of all customers), mostly *sambusa* and *piroshky*; main dishes (19.0%), mostly meat dishes (*plov* and *shashlik*); and sweet pastries and confectionery (17.9%), mostly cookies, buns and candies. The most frequently purchased beverages were tea and coffee (11.3% of all customers), soft drinks and juices (9.8%) and non-alcoholic fermented traditional beverages (4.6%). Fruit and milk were the least frequently purchased food items (1.1% and 0.4% of all customers, respectively). Frequencies of the foods and beverages purchased in each country are presented in Figure 2. Purchases of sweets pastries and confectionery, as well as sandwiches, were more frequently observed in Turkmenistan; bread, fruit, soft drinks and juices in Tajikistan; tea, coffee, traditional beverages and alcoholic beverages in Kyrgyzstan; and water in Kazakhstan. No statistically significant differences amongst cities were found in the frequency of purchase of savoury pastries and snacks, main dishes or milk. Supplementary Table S3 presents the food items purchased by sex, age and weight status of the customers observed.

The median energy content of a street food purchase was 529 kcal, with the highest contribution to the TEV from carbohydrates (56.6%), followed by total fat (33.5%) and protein (12.0%). SFA and TFA median contents were 4.7 g and 0.36 g, corresponding to 21.4% and 16.5% of the maximum daily intake recommendations for these nutrients, respectively. The purchases presented a median sodium content of 745 mg, which accounted for 37.3% of its maximum daily intake recommendation, while supplying 304 mg of potassium (8.7% of its minimum recommendation). The median energy density was 308 kcal/100 g, ranging from 274/275 kcal/100 g in Tajikistan/Kyrgyzstan to 432 kcal/100 g in Turkmenistan. Purchases presented similar median densities of SFA (2.7 g/100 g), MUFA (2.8 g/100 g) and PUFA (2.7 g/100 g), and median TFA ranged from 0.08 g/100 g in Tajikistan to 0.32 g/100 g in Kazakhstan. The median sodium to potassium ratio was higher than 1 in all settings, ranging from 4.3 in Kazakhstan to 6.6 in Tajikistan (Table 2). Purchases containing at least one industrial food item showed higher energy density (518 vs. 286 kcal/100 g, *p* < 0.001), SFA (5.6 vs. 2.6 g/100 g, *p* < 0.001) and TFA contents per 100 g (0.23 vs. 0.19 g/100 g, *p* = 0.001) than those with no industrial food items.

Table 3 presents the estimated nutritional composition of the purchases by sex, age and weight status of the customers. Men presented purchases significantly richer in energy, protein, total fat, SFA, MUFA, TFA and sodium. No differences regarding sex were found for the nutritional composition per 100 g of purchase. Purchases from younger customers had a higher content of TFA, sodium and sodium to potassium ratio; while purchases from older customers showed higher energy density and higher contents per 100 g of protein and carbohydrates. Overweight/obese customers presented purchases with higher energy, total fat and TFA contents. Considering nutrient density (per 100 g), purchases from overweight/obese customers were also richer in energy, carbohydrates, PUFA, *n*−6 and sodium.

## 4. Discussion

This study reports on relevant and innovative data regarding street food purchase and nutritional composition in four urban areas of Central Asia. Frequent purchase of street food was observed, demonstrated by the large number of food items bought within a limited observation period, and a wide variety of ready-to-eat foods and beverages was identified.

Traditional foods, such as *sambusa* (pastry with a savoury filling, usually meat or vegetables and spices), *lepyoshka* (traditional bread) and local main dishes, as well as tea and non-alcoholic fermented beverages, were commonly bought, indicating that local and traditional foods and beverages are important for the daily food consumption in these populations. However, it is noteworthy that almost one-third of the customers bought industrial foods items, mostly soft drinks, cookies and candies; it was also observed a high level of purchase of fried savoury pastries and meat-based dishes, coupled with a very low frequency of purchase of fruit. These results suggest that street food purchases in these settings have been directed towards a more Westernized consumption pattern, which is consistent with the nutrition transition process currently occurring in LMIC [3,30]. In *Almaty* and *Ashgabat*, the frequency of purchase of industrial foods and beverages was particularly concerning since this type of food items are usually energy-dense and rich in fat, SFA and TFA, sugars and salt [31]. In this study, we observed that the purchases containing at least one industrial food item showed higher energy density, SFA and TFA contents. The consumption of ultra-processed foods has been linked to a lower quality of the diet [32,33], and higher prevalence of obesity and other diet-related NCD [34,35,36], which makes urgent the implementation of efforts including information campaigns to raise consumers’ awareness regarding the importance of choosing nutritional dense non-processed foods, as well as behaviour interventions to shape better food choices.

Overall, the median energy content of a street food purchase was 529 kcal; in all four cities, the highest contribution for TEV came from carbohydrates, followed by fat and protein, which was also reported in other settings [37,38,39]. A street food purchase provided considerable amounts of SFA and TFA, exceeding 40% of the WHO daily maximum recommendation for SFA [26] in Turkmenistan, and almost 30% of the WHO daily maximum recommendation for TFA [27] in Kazakhstan. It was also observed that, from a single street food purchase, almost one in every three customers (29.6%) exceeded 50% of the maximum recommendations for SFA, and one in every five (20.8%) exceeded 50% of the maximum recommendation for TFA. Both homemade and industrial foods have been identified as important sources of TFA in the street food context of these settings [10,11,12,13]. Another work in urban Delhi, India, showed high contents of total fat, SFA and TFA in street foods and oils used by street vendors [40]. High intakes of *trans* fats have been extensively associated with all-cause mortality and cardiovascular disease [41], and some countries in the WHO European Region have introduced legislation to limit its content in the food supply to a maximum of 2 g/100 g of fat [42]. The Eurasian Economic Commission adopted similar actions [43], enforcing Tajikistan, Kyrgyzstan and Kazakhstan to align with these practices up to 2018. However, according to our results, almost half (48.7%) of the industrial food items purchased with TFA information exceeded this limit, showing that progress must be made in order to achieve this goal, and repeated monitoring would be necessary to ensure compliance. Furthermore, high TFA levels in homemade foods suggest that its preparation commonly may include unhealthy cooking methods, such as frying, as well as the use of TFA-rich fats and shortenings. As such, to raise awareness of the street vendors for the importance of improving the quantity and quality of the fats added during preparation and cooking may help to reduce saturated and *trans* fats in homemade foods. Governmental support to street vendors and manufacturers for the substitution to healthier fats and improvement of the lipid profile of the food products should also be discussed.

Our results also showed that one single purchase provided more than one-third of the maximum daily intake recommendation for sodium [28]; furthermore, almost one-quarter (22.4%) of the customers exceeded its limit of 2000 mg in just one purchase. Sodium to potassium ratio exceeded four times the recommendation [28,29] in purchases from all settings, being of even greater concern in the urban contexts of *Dushanbe* and *Bishkek*, in line with previous evidence [44]. The homemade food items purchased presented a median sodium to potassium ratio significantly higher than the industrial ones (5.20 vs. 3.29, *p* < 0.001), which could be attributed to the excessive use of added salt and/or sodium-rich ingredients (e.g., soy sauce) during homemade foods’ preparation/cooking, as observed in other settings [45]. However, foods classified as homemade or industrial are heterogeneous in terms of the groups they belong to, and some are not sources of salt. Therefore, direct comparisons should be conducted with caution. For example, some food items classified as industrial (e.g., confectionery, soft drinks and juices), which usually have very low sodium contents, may contribute to explain these differences. The high sodium contents found in certain homemade foods may also reflect the influence of the “Silk Road” pattern, in which the use of salt as a food preservative remains a common tradition in Central Asian countries [46]. In 2010, diets high in sodium were among the leading risk factors for disease in Central Asia [47], and current evidence suggests that a reduction in dietary sodium decreases cardiovascular morbidity and mortality [48]. Public health strategies could include targets for salt reduction and/or mandatory maximum limits for industrial products, as well as the development of programmes targeting both vendors and consumers, to raise their awareness about the health hazards associated with adding too much salt to foods. Furthermore, and despite the protective effect of dietary potassium on cardiovascular disease risk [49], the street food purchases assessed in this study appeared to be a very poor source of this nutrient, in part due to a vestigial level of purchase of ready-to-eat fruits and vegetables by the customers observed. Results from the FEEDcities project have been clearly showing that availability of street-vended fruit is very low in all settings studied [10,11,12,13]. However, it should be pointed out that vending sites selling exclusively non-prepared fresh fruits and vegetables were excluded from this study, which may have underestimated the purchases of fruits and vegetables. However, this exclusion criterion was defined because these venues were mostly directed towards household food consumption, and not for immediate consumption, as the definition of ‘street food’ as ‘ready-to-eat food’ implies. Strategies aimed at increasing the availability of ready-to-eat fruits or improving the amounts of vegetables in some main dishes traditionally containing them as ingredients (e.g., *plov*, *lagman*), can help improve the consumption of these foods, which are nutrient-dense, rich in potassium and with low sodium content.

Regarding customers’ characteristics, the street food purchases made by men and by overweight or obese customers presented a less healthy nutritional profile, showing in general higher levels of energy, fat (total, saturated and/or *trans*), sodium and sodium to potassium ratio than women and normal-weighted customers. Purchases from younger customers were also richer in *trans*-fat and sodium. These results can be useful in efforts to design targeted interventions towards diet-related NCD prevention.

Some limitations of this study should be acknowledged. Customers’ weight status was estimated using direct observation, which is not yet validated for these populations. Although body measurement is the established gold standard, this method would bring a number of problems to achievable collection of data, namely: (1) the need for material and human resources; (2) large apparatus around the vendors, disturbing their activity and possibly causing the alteration of the customers’ behaviour; and (3) cultural barriers to body measurement. A faster, more practical and culture-friendly approach to collect accurate anthropometric data in those settings has become essential. Self-report of height and weight has been extensively used; however, under-reporting of weight and over-reporting of height, leading to under-estimation of body mass index, have been documented [50]. As such, the validation of the estimation of weight status by direct observation by trained professionals in less developed contexts would be an important step in future work. Direct observation of purchases without interviewing also implies that we cannot ensure that all customers bought food items only for themselves, and for one single meal. However, the number of food items purchased per customer, which was close to one, suggested that this assumption should be correct in the majority of cases.

Another limitation of the present research was the inability to discriminate sugars within carbohydrates. Nonetheless, it was observed that a significant proportion (27.6%) of our sample purchased sugar-rich food items, namely sweet pastries, confectionery, soft drinks and industrial juices. In line with this, frequent availability of soft drinks and other sugary foods in the streets of these cities was previously reported [10,11,12,13]. High intake of free sugars increases overall energy intake, leading to weight gain and increased risk of diet-related NCD [51,52], and WHO recommends reducing its intake to less than 10% of TEV [28]. As such, future work on the content of free sugars in the street foods available in these settings is recommended.

Among the strengths of this study, we highlight the collection of consumption data through observation of customers at distance rather than interview, which allowed us to minimize potential interference and, thus, potential behavioural or social desirability biases. A high level of concordance between observers, both on demographic and anthropometric characteristics of the customers, as well as on the food items purchased and their quantities, was observed. This was due to successful training, standardization of procedures and constant supervision, and resulted in reproducible observational data on street food purchase and customers’ characteristics. Regarding nutritional composition, not only energy and macronutrients content was analysed, but also the lipid profile, as well as sodium and potassium contents. This represents a novel approach in relation to other studies on street food, providing a much more detailed view of the key nutrients for diet-related chronic disease. However, nutritional contents per 100 g may be diluted by the water content of the beverages that were purchased together with the foods, although we estimate that this effect should be minor, as only 9.9% of customers purchased foods with beverages (as shown in Table 1). However, these results should be interpreted carefully and in association with the contents per serving. Regarding fatty acids contents estimation (SFA, PUFA, *n*−3, *n*−6, MUFA and TFA), the lack of available data in the food composition tables and food labels used may have generated some error associated with the estimates of those specific nutrients. However, we assume that this error should be limited, since the majority of the purchases assessed (*n* = 494, 87.6%) had data available from chemical analyses. The inclusion of fatty acids data in both food composition tables and food labels would be a valuable contribution not only to research and policy, but also to the improvement of the information provided to the consumers. Finally, although generalizability is limited due to local cultural specificities, the FEEDCities methodology allows for standardized assessment, resulting in comparable results among different cities or countries.

## 5. Conclusions

In conclusion, street food purchase in these urban areas of Central Asia is frequent and characterized by the acquisition of local traditional foods and beverages concomitantly with industrialized food products. In general, street food purchases were energy-dense and rich in sodium, saturated and *trans* fats, and poor in potassium, which in the long term can have a negative impact on health. Further research on the prevalence, frequency and determinants of street food consumption in the general population, as well as practices among vendors, ingredients used and its determinants, using complementary methodology such as interviews, would contribute to deepen the knowledge regarding this issue. Our findings reinforce the importance of implementing public health strategies on healthier diets promotion and NCD prevention, on several fronts, such as public awareness, legislation, product reformulation and labelling improvement. Such policies could be integrated in existing national programmes on NCD, food security and nutrition, emphasizing mostly on sodium reduction, improvement of lipid profile and *trans* fat elimination.

## Figures and Tables

**Table 1:** Demographic and anthropometric characteristics and items purchased by the customers observed in street food vending sites of Dushanbe (Tajikistan), Bishkek (Kyrgyzstan), Ashgabat (Turkmenistan) and Almaty (Kazakhstan).

	Total *n* = 714	Dushanbe *n* = 81	Bishkek *n* = 237	Ashgabat *n* = 257	Almaty *n* = 139	*p*
	*n* (%)					
Sex						
Male	310 (43.4)	53 (34.6)	96 (40.5)	119 (46.3)	42 (30.2)	**<0.001**
Female	404 (56.6)	28 (65.4)	141 (59.5)	138 (53.7)	97 (69.8)	
Age ^a^						
<35 years	286 (44.5)	38 (56.7)	101 (47.9)	97 (41.8)	50 (37.9)	**0.045**
≥35 years	356 (55.5)	29 (43.3)	110 (52.1)	135 (58.2)	82 (62.1)	
Weight status ^a^						
Underweight/normal weight	503 (76.7)	52 (76.5)	178 (82.8)	178 (73.6)	95 (72.5)	0.069
Overweight/obesity	153 (23.3)	16 (23.5)	37 (17.2)	64 (26.4)	36 (27.5)	
Number of items purchased						
1 (median)	595 (83.3)	66 (81.5)	203 (85.7)	213 (82.9)	113 (81.3)	0.666
>1	119 (16.7)	15 (18.5)	34 (14.3)	44 (17.1)	26 (18.7)	
Purchased foods or beverages?						
Only foods	493 (69.1)	55 (67.9)	131 (55.3)	222 (86.4)	85 (61.2)	**<0.001**
Only beverages	150 (21.0)	19 (23.5)	79 (33.3)	15 (5.8)	37 (26.6)	
Foods and beverages	71 (9.9)	7 (8.6)	27 (11.4)	20 (7.8)	17 (12.2)	
Purchased homemade or industrial?						
Only homemade	486 (68.1)	58 (71.6)	175 (73.8)	174 (67.7)	79 (56.8)	**0.003**
Only industrial	201 (28.1)	23 (28.4)	58 (24.5)	71 (27.6)	49 (35.3)	
Homemade and industrial	27 (3.8)	0 (0.0)	4 (1.7)	12 (4.7)	11 (7.9)	

Values in bold represent statistically significant differences according to Pearson’s Chi-squared test with a significance level of 0.05. ^a^ For the variables age and weight status, the data presented corresponds to the customers in which there was agreement between observers (total: *n* = 642 and *n* = 656; Tajikistan: *n* = 67 and *n* = 68; Kyrgyzstan: *n* = 211 and *n* = 215; Turkmenistan: *n* = 232 and *n* = 242; Kazakhstan: *n* = 132 and *n* = 131).

**Table 2:** Estimated nutritional composition of the purchases made by the street food customers observed in Dushanbe (Tajikistan), Bishkek (Kyrgyzstan), Ashgabat (Turkmenistan) and Almaty (Kazakhstan).

	Total (*n* = 564 ^a^)	Dushanbe (*n* = 62 ^a^)	Bishkek (*n* = 158 ^a^)	Ashgabat (*n* = 242 ^a^)	Almaty (*n* = 102 ^a^)	*p*
	Median (P25–P75)					
Amount purchased (g)	120 (95–278)	120 (58–281)	176 (100–306)	120 (99–265)	103 (90–296)	0.062
Per total purchase						
Energy (kcal)	529 (329–850)	352 (311–816)	451 (314–615)	568 (343–993)	557 (326–915)	**<0.001**
Protein (g)	13.4 (8.5–25.6)	12.7 (8.6–23.7)	18.1 (10.6–23.4)	13.2 (7.2–29.7)	13.5 (7.0–25.5)	0.267
CHO (g)	68.1 (42.6–107.6)	69.1 (33.0–91.3)	60.0 (44.7–73.9)	70.5 (54.5–130.3)	69.5 (40.3–101.8)	**<0.001**
Total fat (g)	20.1 (5.3–32.6)	11.1 (0.9–25.6)	16.2 (5.3–28.8)	22.8 (9.0–44.4)	21.5 (7.9–40.5)	**<0.001**
SFA (g) ^b^	4.7 (1.2–12.4)	2.5 (0.5–9.9)	3.4 (1.1–12.3)	9.0 (1.1–15.7)	4.6 (1.7–11.1)	**<0.001**
MUFA (g) ^b^	5.3 (1.6–9.4)	3.7 (0.3–7.9)	3.8 (1.4–9.3)	5.9 (2.0–11.3)	5.3 (2.5–10.7)	**<0.001**
PUFA (g) ^b^	5.0 (1.3–9.1)	2.9 (0.2–9.6)	4.0 (0.7–7.8)	6.5 (1.7–9.8)	4.2 (1.9–8.9)	**<0.001**
*n*−6 (g) ^b^	4.3 (1.2–8.6)	2.7 (0.2–8.8)	3.9 (0.6–7.6)	6.3 (1.4–9.5)	3.6 (1.9–8.9)	**<0.001**
*n*−3 (g) ^b^	0.1 (0.0–0.2)	0.0 (0.0–0.1)	0.1 (0.0–0.4)	0.1 (0.1–0.3)	0.1 (0.0–0.2)	**0.002**
TFA (g) ^b^	0.36 (0.06–0.86)	0.08 (0.02–0.38)	0.29 (0.02–1.38)	0.36 (0.06–0.55)	0.60 (0.20–2.14)	**<0.001**
Na (mg) ^c^	745 (475–1783)	723 (479–1708)	861 (561–1411)	851 (477–2314)	681 (316–2085)	0.209
K (mg) ^d^	304 (180–713)	226 (121–527)	282 (149–571)	414 (245–850)	288 (174–651)	**<0.001**
Per 100 g of purchase						
Energy (kcal)	308 (212–605)	274 (174–484)	275 (158–419)	432 (276–663)	315 (212–621)	**<0.001**
Protein (g)	10.6 (5.5–17.2)	10.0 (6.0–14.1)	10.6 (5.5–14.8)	11.0 (5.4–19.5)	8.9 (5.6–16.9)	0.154
CHO (g)	53.5 (25.2–74.8)	49.9 (25.2–58.9)	31.0 (17.3–57.6)	56.8 (29.6–108.9)	42.8 (30.2–75.2)	**<0.001**
Total fat (g)	10.1 (3.3–22.7)	5.2 (1.8–14.9)	7.7 (2.3–14.9)	17.3 (4.1–30.1)	11.4 (4.7–24.1)	**<0.001**
SFA (g) ^b^	2.7 (0.7–7.2)	1.8 (0.7–4.3)	2.1 (0.4–5.2)	3.9 (0.7–11.0)	2.7 (1.4–7.9)	**<0.001**
MUFA (g) ^b^	2.8 (0.9–6.8)	1.5 (0.6–4.7)	2.2 (0.7–3.9)	4.1 (1.2–9.6)	3.2 (1.4–6.8)	**<0.001**
PUFA (g) ^b^	2.7 (1.1–6.4)	1.5 (0.3–5.0)	1.5 (0.8–3.8)	3.6 (1.3–7.1)	3.7 (1.5–6.7)	**<0.001**
*n*−6 (g) ^b^	2.7 (1.0–6.0)	1.3 (0.3–4.1)	1.4 (0.7–3.4)	3.5 (1.2–6.7)	3.6 (1.2–6.5)	**<0.001**
*n*−3 (g) ^b^	0.1 (0.0–0.2)	0.0 (0.0–0.1)	0.1 (0.0–0.1)	0.1 (0.0–0.2)	0.1 (0.0–0.2)	**<0.001**
TFA (g) ^b^	0.21 (0.05–0.51)	0.08 (0.03–0.15)	0.22 (0.02–0.55)	0.21 (0.04–0.46)	0.32 (0.18–1.45)	**<0.001**
Na (mg) ^c^	514 (411–1042)	467 (348–657)	508 (325–740)	611 (467–1216)	519 (324–1027)	**0.005**
K (mg) ^d^	247 (150–474)	145 (114–250)	205 (124–303)	328 (150–631)	286 (166–461)	**<0.001**
Na/K ratio ^d^	4.7 (2.7–6.3)	6.6 (4.1–8.0)	5.2 (3.1–8.7)	4.4 (2.6–5.6)	4.3 (2.3–5.6)	**<0.001**

CHO, carbohydrates; SFA, saturated fatty acids; MUFA, monounsaturated fatty acids; PUFA, polyunsaturated fatty acids; TFA, trans fatty acids; Na, sodium; K, potassium. Values in bold represent statistically significant differences according to Kruskal–Wallis test with a significance level of 0.05. ^a^ Data on customers purchasing only beverages were not included. ^b^ For these variables, the sample size is lower (*n* = 494) due to missing values. ^c^ For this variable, the sample size is lower (*n* = 473) due to missing values. ^d^ For these variables, the sample size is lower (*n* = 457) due to missing values.

**Table 3:** Estimated nutritional composition of the purchases made by the street food customers observed in Dushanbe (Tajikistan), Bishkek (Kyrgyzstan), Ashgabat (Turkmenistan) and Almaty (Kazakhstan), by sex, age and weight status.

	Sex			Age			Weight Status		
	Male (*n* = 242 ^a^)	Female (*n* = 322 ^a^)	*p*	<35 years (*n* = 226 ^a^)	≥35 years (*n* = 281 ^a^)	*p*	Underweight /Normal Weight (*n* = 398 ^a^)	Overweight /Obesity (*n* = 122 ^a^)	*p*
	Median (P25–P75)			Median (P25–P75)			Median (P25–P75)		
Amount purchased (g)	120 (96–317)	120 (90–265)	0.051	132 (96–296)	120 (68.1–265.0)	**0.029**	120 (96–278)	120 (81–265)	0.411
Per total purchase									
Energy (kcal)	555 (337–930)	509 (311–717)	**0.018**	500 (327–893)	557 (329–750)	0.957	488 (324–730)	570 (343–978)	**0.020**
Protein (g)	15.9 (9.6–30.7)	13.2 (7.6–13.2)	**0.004**	15.3 (8.6–26.2)	13.2 (8.5–24.2)	0.292	13.2 (8.5–24.2)	13.6 (8.5–26.2)	0.529
CHO (g)	68.1 (45.1–122.3)	68.1 (40.5–100.1)	0.271	68.1 (41.5–118.3)	68.1 (42.8–100.1)	0.992	68.1 (41.5–100.1)	72.3 (49.3–122.3)	0.051
Total fat (g)	22.8 (6.5–35.2)	16.0 (5.3–30.9)	**0.002**	20.9 (7.6–35.1)	18.5 (5.2–31.8)	0.317	19.4 (5.3–31.2)	22.8 (6.3–43.9)	**0.006**
SFA (g) ^b^	7.3 (1.5–12.8)	3.2 (1.1–11.1)	**0.025**	5.8 (1.5–12.3)	3.7 (1.0–12.3)	0.149	4.6 (1.1–12.3)	4.6 (1.3–12.8)	0.475
MUFA (g) ^b^	6.3 (1.7–9.7)	4.4 (1.5–9.3)	**0.041**	5.9 (1.9–9.5)	4.4 (1.1–9.3)	0.119	4.9 (1.6–9.3)	5.7 (1.5–11.0)	0.237
PUFA (g) ^b^	5.6 (1.3–9.5)	4.5 (1.3–8.4)	0.429	5.6 (1.4–9.6)	4.0 (1.3–8.3)	0.111	4.5 (1.3–9.1)	5.2 (1.4–9.6)	0.441
*n*−6 (g) ^b^	4.7 (1.2–9.0)	4.2 (1.3–8.3)	0.559	5.1 (1.2–9.5)	3.5 (1.2–7.6)	0.126	4.3 (1.2–8.4)	3.9 (1.4–9.5)	0.471
*n*−3 (g) ^b^	0.1 (0.1–0.3)	0.1 (0.0–0.2)	0.105	0.1 (0.1–0.3)	0.1 (0.0–0.2)	0.120	0.1 (0.0–0.2)	0.1 (0.0–0.3)	0.891
TFA (g) ^b^	0.38 (0.05–1.15)	0.31 (0.06–0.67)	**0.002**	0.38 (0.11–0.86)	0.32 (0.03–0.76)	**0.036**	0.31 (0.05–0.73)	0.40 (0.06–1.00)	**0.025**
Na (mg) ^c^	906 (560–2165)	672 (470–1687)	**0.030**	1062 (560–1943)	561 (416–1653)	**0.001**	753 (470–1750)	709 (470–2042)	0.937
K (mg) ^d^	343 (180–718)	291 (180–691)	0.239	340 (180–777)	250 (175–648)	0.078	313 (180–691)	288 (180–778)	0.931
Per 100 g of purchase									
Energy (kcal)	286 (210–552)	314 (212–638)	0.370	287 (185–531)	314 (228–663)	**0.016**	306 (210–552)	382 (253–674)	**0.025**
Protein (g)	10.6 (5.5–17.1)	10.6 (5.5–17.4)	0.929	8.7 (5.2–15.0)	11.0 (6.4–18.2)	**0.003**	10.6 (5.4–17.1)	10.8 (6.5–19.5)	0.132
CHO (g)	53.5 (24.2–67.4)	53.1 (25.5–77.1)	0.325	40.2 (20.6–63.7)	56.8 (26.3–106.9)	**<0.001**	50.9 (22.7–70.5)	56.8 (29.6–101.8)	**0.030**
Total fat (g)	9.3 (3.5–24.1)	10.8 (3.2–20.1)	0.926	10.0 (3.7–20.9)	10.0 (3.3–24.0)	0.554	10.0 (2.5–20.1)	13.2 (4.3–30.1)	0.065
SFA (g) ^b^	3.2 (0.7–9.0)	2.6 (0.7–7.0)	0.277	3.0 (1.3–7.1)	2.6 (0.7–8.9)	0.438	2.7 (0.7–7.1)	2.7 (0.7–10.9)	0.322
MUFA (g) ^b^	2.6 (0.9–7.4)	3.0 (0.9–6.1)	0.619	2.8 (1.1–6.4)	2.5 (0.7–7.1)	0.971	2.8 (0.8–6.3)	3.4 (1.1–10.7)	0.111
PUFA (g) ^b^	2.6 (1.1–5.6)	3.0 (1.1–6.6)	0.645	2.6 (0.9–5.4)	2.7 (1.1–6.4)	0.229	2.6 (1.1–5.8)	3.8 (1.3–6.6)	**0.023**
*n*−6 (g) ^b^	2.5 (1.0–5.3)	2.7 (1.0–6.5)	0.572	2.4 (0.8–5.0)	2.7 (1.0–6.3)	0.243	2.5 (0.9–5.6)	3.3 (1.2–6.6)	**0.032**
*n*−3 (g) ^b^	0.1 (0.0–0.2)	0.1 (0.0–0.1)	0.210	0.1 (0.0–0.1)	0.1 (0.0–0.2)	0.773	0.1 (0.0–0.1)	0.1 (0.0–0.2)	0.368
TFA (g) ^b^	0.21 (0.04–0.57)	0.20 (0.05–0.48)	0.752	0.21 (0.08–0.46)	0.19 (0.03–0.52)	0.593	0.21 (0.05–0.46)	0.25 (0.04–0.76)	0.219
Na (mg) ^c^	505 (412–1042)	521 (411–1065)	0.980	484 (345–1064)	525 (435–1042)	0.377	490 (388–1042)	620 (467–1136)	**0.045**
K (mg) ^d^	246 (145–500)	249 (150–461)	0.337	244 (145–474)	264 (150–461)	0.266	244 (150–454)	305 (166–500)	0.060
Na/K ratio ^d^	5.3 (3.1–6.3)	4.3 (2.6–5.9)	0.141	5.3 (2.7–6.6)	4.2 (3.0–5.8)	**0.046**	4.9 (2.6–6.3)	4.2 (3.0–5.8)	0.604

CHO, carbohydrates; SFA, saturated fatty acids; MUFA, monounsaturated fatty acids; PUFA, polyunsaturated fatty acids; TFA, *trans* fatty acids; Na, sodium; K, potassium. Values in bold represent statistically significant differences according to the Mann–Whitney’s U test with a significance level of 0.05. ^a^ Data on customers purchasing only beverages were not included. In addition, regarding age and weight status, data presented corresponds to the customers in which there was agreement between observers. ^b^ For these variables, the sample size is lower (male: *n* = 206; female: *n* = 288; <35 years: *n* = 198; ≥35 years: *n* = 247; underweight/normal weight: *n* = 345; overweight/obese: *n* = 107) due to missing values. ^c^ For this variable, the sample size is lower (male: *n* = 200; female: *n* = 273; <35 years: *n* = 185; ≥35 years: *n* = 236; underweight/normal weight: *n* = 328; overweight/obese: *n* = 103) due to missing values. ^d^ For these variables, the sample size is lower (male: *n* = 191; female: *n* = 266; <35 years: *n* = 175; ≥35 years: *n* = 230; underweight/normal weight: *n* = 315; overweight/obese: *n* = 101) due to missing values.

## Data Availability

The data presented in this study are available upon request to the corresponding author.

## Supplementary Materials

The following are available online at https://www.mdpi.com/article/10.3390/nu13103651/s1, Table S1: Inter-observer concordance on demographic and anthropometric characteristics and items purchased by the customers observed in street food vending sites of Dushanbe (Tajikistan), Bishkek (Kyrgyzstan), Ashgabat (Turkmenistan) and Almaty (Kazakhstan). Table S2: Decision rules for the elimination of conflicts of observation regarding food items and quantities purchased. Table S3: Ready-to-eat foods and beverages purchased by the street food customers observed in Dushanbe (Tajikistan), Bishkek (Kyrgyzstan), Ashgabat (Turkmenistan) and Almaty (Kazakhstan), overall and by sex, age and weight status.
