# Supplementary material for: A Cross-Sectional Study of the Street Foods Purchased by Customers in Urban Areas of Central Asia
Source: Nutrients. 2021 Oct 19;13(10):3651. doi: 10.3390/nu13103651 (PMC8539089; doi:10.3390/nu13103651)
Supplement: Supplementary file 1 [file nutrients-13-03651-s001.zip › nutrients-1383386-supplementary.pdf]

Supplementary Materials

Table S1. Inter-observer concordance on demographic and anthropometric characteristics and items purchased by the customers observed in street food vending sites of *Dushanbe* (Tajikistan), *Bishkek* (Kyrgyzstan), *Ashgabat* (Turkmenistan) and *Almaty* (Kazakhstan).

	**Overall**		**Tajikistan**		**Kyrgyzstan**		**Turkmenistan**		**Kazakhstan**	
	**% agreement**	**Cohen's Kappa (95% CI)**	**% agreement**	**Cohen's Kappa (95% CI)**	**% agreement**	**Cohen's Kappa (95% CI)**	**% agreement**	**Cohen's Kappa (95% CI)**	**% agreement**	**Cohen's Kappa (95% CI)**
**CUSTOMERS**	**n=714**		**n=81**		**n=237**		**n=257**		**n=139**	
**Sex**										
Male<br>Female	100.0%	1.00 (0.93-1.00)	100.0%	1.00 (0.78-1.00)	100.0%	1.00 (0.87-1.00)	100.0%	1.00 (0.88-1.00)	100.0%	1.00 (0.83-1.00)
**Age**										
<35 years<br>≥35 years	89.9%	0.80 (0.72-0.87)	82.7%	0.65 (0.44-0.87)	89.0%	0.78 (0.65-0.91)	90.3%	0.80 (0.68-0.92)	94.7%	0.89 (0.73-1.00)
**Weight status**										
Underweight/Normal weight<br>Overweight/obesity	91.9%	0.79 (0.71-0.86)	84.0%	0.60 (0.39-0.82)	90.7%	0.71 (0.59-0.84)	94.2%	0.86 (0.73-0.98)	94.2%	0.86 (0.69-1.00)
**Purchased at least one food**										
Yes<br>No	100.0%	1.00 (0.93-1.00)	100.0%	1.00 (0.78-1.00)	100.0%	1.00 (0.87-1.00)	100.0%	1.00 (0.89-1.00)	100.0%	1.00 (0.83-1.00)
**Purchased at least one beverage**										
Yes<br>No	99.2%	0.98 (0.91-1.00)	95.1%	0.88 (0.66-1.00)	99.2%	0.98 (0.86-1.00)	100.0%	1.00 (0.88-1.00)	100.0%	1.00 (0.83-1.00)
**Number of items purchased**										
One (median)<br>More than one	99.7%	0.99 (0.93-1.00)	98.8%	0.96 (0.74-1.00)	100.0%	1.00 (0.87-1.00)	99.6%	0.99 (0.86-1.00)	100.0%	1.00 (0.83-1.00)
**ITEMS**	**n=852**		**n=101**		**n=276**		**n=310**		**n=165**	
**Item purchased**	97.6%	0.98 (0.97-0.99)	97.9%	0.98 (0.93-1.00)	94.5%	0.94 (0.92-0.96)	99.7%	1.00 (0.97-1.00)	98.8%	0.99 (0.96-1.00)
**Quantity purchased**	95.3%	0.94 (0.92-0.97)	92.6%	0.89 (0.79-0.99)	94.5%	0.93 (0.89-0.98)	96.8%	0.96 (0.92-1.00)	95.2%	0.94 (0.87-1.00)

95% CI: 95% confidence interval.

Table S2. Decision rules for the elimination of conflicts of observation regarding food items and quantities purchased.

Type of disagreement	Criteria used	Example
**Disagreements on the food item purchased**		
The two observers registered two different food items (n=5).	Conflicting food items were checked for its availability on the corresponding vending site.*	Observer A registered "coffee" and observer B registered "tea". The corresponding vending site did not have coffee available. It was assumed that the customer purchased tea.
The two observers registered the same food item, but with different degrees of specificity (n=15).	The broadest observation was assumed.	Observer A registered "samosa" and observer B registered "chicken samosa". It was assumed that the customer purchased samosa.
The two observers registered a different number of food items (n=10).	The most complete observation was assumed, unless the conflicting item was not available in the corresponding vending site.	Observer A registered "samosa" and observer B registered "samosa" and "tea". It was assumed that the customer purchased samosa and tea. If the corresponding vending site did not have tea available, it would be assumed that the customer purchased samosa.
**Disagreements on the quantity purchased**		
The two observers registered the same food item, but different quantities (n=40).	The average quantity between the two observations was assumed.	Observer A registered 200mL of tea and observer B registered 300mL of tea. It was assumed that the customer purchased 250mL of tea.

*Observations should be excluded in the cases where the availability criterion does not solve the conflict: (1) if the conflicting food items are both available at the corresponding vending site; or (2) if none of the conflicting food items are available at the corresponding vending site. These particular situations did not occur.

Table S3. Ready-to-eat foods and beverages purchased by the street food customers observed in *Dushanbe* (Tajikistan), *Bishkek* (Kyrgyzstan), *Ashgabat* (Turkmenistan) and *Almaty* (Kazakhstan), overall and by sex, age and weight status.

	Total		Sex					Age[a]					Weight status[a]				
			Male		Female		p	<35 years		≥35 years		p	Underweight /normal		Overweight /obesity		p
	n	%	n	%	n	%		n	%	n	%		n	%	n	%	
**Tajikistan**	n=81		n=53		n=28			n=38		n=29			n=52		n=16		
**Foods[b]**	62	76.5	40	75.5	22	78.6	0.754	31	81.6	21	72.4	0.373	41	78.8	10	62.5	0.187
Savoury pastries and snacks	21	25.9	13	24.5	8	28.6	0.693	13	34.2	3	10.3	**0.023**	15	28.8	2	12.5	0.187
Main dishes	9	11.1	5	9.4	4	14.3	0.509	4	10.5	5	17.2	0.425	4	7.7	2	12.5	0.553
Sweet pastries and confectionery	10	12.4	8	15.1	2	7.2	0.301	8	21.1	2	6.9	0.107	9	17.3	0	0.0	0.074
Breads	21	25.9	13	24.5	8	28.6	0.693	7	18.4	13	44.8	**0.019**	10	19.2	7	43.8	**0.048**
Sandwiches	1	1.2	1	1.9	0	0.0	0.465	1	2.6	0	0.0	0.379	1	1.9	0	0.0	0.576
Fruit	4	4.9	3	5.7	1	3.6	0.680	0	0.0	0	0.0	-	4	7.7	0	0.0	0.253
**Beverages[b]**	26	32.1	18	34.0	8	28.6	0.621	13	34.2	8	27.6	0.562	16	30.8	8	50.0	0.159
Tea and coffee	4	4.9	2	3.8	2	7.1	0.506	4	10.5	0	0.0	0.072	4	7.7	0	0.0	0.253
Soft drinks and juices	15	18.5	11	20.8	4	14.3	0.476	6	15.8	6	20.7	0.604	7	13.5	7	43.8	**0.009**
Fermented traditional beverages[c]	2	2.5	2	3.8	0	0.0	0.298	2	5.3	0	0.0	0.210	2	3.8	0	0.0	0.426
Water	3	3.7	1	1.9	2	7.1	0.234	1	2.6	1	3.4	0.846	2	3.8	0	0.0	0.426
Alcoholic beverages[d]	2	2.5	2	3.8	0	0.0	0.298	0	0.0	1	3.4	0.249	1	1.9	1	6.3	0.370
Milk	0	0.0	0	0.0	0	0.0	-	0	0.0	0	0.0	-	0	0.0	0	0.0	-
**Kyrgyzstan**	n=237		n=96		n=141			n=101		n=110			n=178		n=37		
**Foods[b]**	158	66.7	59	61.5	99	70.2	0.160	68	67.3	72	65.5	0.774	121	68.0	25	67.6	0.961
Savoury pastries and snacks	56	23.6	19	19.8	37	26.2	0.251	24	23.8	26	23.6	0.983	42	23.6	11	29.7	0.431
Main dishes	48	20.3	22	22.9	26	18.4	0.400	24	23.8	17	15.5	0.128	42	23.6	3	8.1	**0.035**
Sweet pastries and confectionery	22	9.3	5	5.2	17	12.1	0.075	9	8.9	12	10.9	0.628	13	7.3	8	21.6	**0.008**
Breads	22	9.3	6	6.3	16	11.3	0.184	4	4.0	14	12.7	**0.023**	17	9.6	3	8.1	0.783
Sandwiches	16	6.8	9	9.4	7	5.0	0.184	10	9.9	5	4.5	0.130	12	6.7	1	2.7	0.348
Fruit	0	0.0	0	0.0	0	0.0	-	0	0.0	0	0.0	-	0	0.0	0	0.0	-
**Beverages[b]**	106	44.7	51	53.1	55	39.0	**0.032**	47	46.5	50	45.5	0.875	78	43.8	16	43.2	0.949
Tea and coffee	51	21.5	21	21.9	30	21.3	0.912	23	22.8	24	21.8	0.868	37	20.8	8	21.6	0.910
Soft drinks and juices	24	10.1	13	13.5	11	7.8	0.150	13	12.9	9	8.2	0.266	19	10.7	1	2.7	0.129
Fermented traditional beverages[c]	21	8.9	11	11.5	10	7.1	0.246	8	7.9	13	11.8	0.345	16	9.0	3	8.1	0.864
Water	1	0.4	0	0.0	1	0.7	0.408	1	1.0	0	0.0	0.296	0	0.0	1	2.7	**0.028**
Alcoholic beverages[d]	8	3.4	5	5.2	3	2.1	0.197	2	2.0	4	3.6	0.470	5	2.8	3	8.1	0.121
Milk	1	0.4	1	1.0	0	0.0	0.225	0	0.0	0	0.0	-	1	0.6	0	0.0	0.648
**Turkmenistan**	n=257		n=119		n=138			n=97		n=135			n=178		n=64		
**Foods[b]**	242	94.2	112	94.1	130	94.2	0.977	92	94.8	127	94.1	0.801	167	93.8	60	93.8	0.984
Savoury pastries and snacks	60	23.4	31	26.1	29	21.0	0.341	30	30.9	26	19.3	**0.040**	46	25.8	11	17.1	0.162

Main dishes	49	19.1	18	15.1	31	22.5	0.135	17	17.5	28	20.7	0.541	32	18.0	14	21.9	0.496
Sweet pastries and confectionery	67	26.1	26	21.9	41	29.7	0.152	23	23.7	40	29.6	0.317	44	24.7	19	29.7	0.437
Breads	49	19.1	28	23.5	21	15.2	0.091	15	15.5	29	21.5	0.249	34	19.1	12	18.8	0.951
Sandwiches	25	9.7	10	8.4	15	10.9	0.506	10	10.3	9	6.7	0.318	17	9.6	6	9.4	0.967
Fruit	4	1.6	2	1.7	2	1.5	0.881	2	2.1	2	1.5	0.738	3	1.7	1	1.6	0.947
**Beverages**[b]	35	13.6	19	16.0	16	11.6	0.308	17	17.5	16	11.9	0.222	26	14.6	8	12.5	0.677
Tea and coffee	9	3.5	6	5.0	3	2.2	0.212	4	4.1	5	3.7	0.870	7	3.9	1	1.6	0.363
Soft drinks and juices	21	8.2	12	10.1	9	6.5	0.299	13	13.4	6	4.4	**0.014**	16	9.0	5	7.8	0.774
Fermented traditional beverages[c]	3	1.2	1	0.8	2	1.5	0.650	0	0.0	3	2.2	0.139	1	0.6	2	3.1	0.112
Water	3	1.2	1	0.8	2	1.5	0.650	0	0.0	3	2.2	0.139	3	1.7	0	0.0	0.296
Alcoholic beverages[d]	0	0.0	0	0.0	0	0.0	-	0	0.0	0	0.0	-	0	0.0	0	0.0	-
Milk	0	0.0	0	0.0	0	0.0	-	0	0.0	0	0.0	-	0	0.0	0	0.0	-
**Kazakhstan**	n=139		n=42		n=97			n=50		n=82			n=95		n=36		
**Foods**[b]	102	73.4	31	73.8	71	73.2	0.940	35	70.0	61	74.4	0.583	69	72.6	27	75.0	0.784
Savoury pastries and snacks	29	20.7	6	14.3	23	23.7	0.209	13	26.0	14	17.1	0.217	21	22.1	7	19.4	0.740
Main dishes	30	21.6	12	28.6	18	18.6	0.188	8	16.0	20	24.4	0.253	15	15.8	11	30.6	0.059
Sweet pastries and confectionery	29	20.9	7	16.7	22	22.7	0.423	10	20.0	19	23.2	0.670	24	25.3	5	13.9	0.162
Breads	10	7.2	3	7.1	7	7.2	0.988	1	2.0	7	8.5	0.127	6	6.3	2	5.6	0.871
Sandwiches	5	3.6	4	9.5	1	1.0	**0.014**	4	8.0	1	1.2	**0.048**	3	3.2	2	5.6	0.523
Fruit	0	0.0	0	0.0	0	0.0	-	0	0.0	0	0.0	-	0	0.0	0	0.0	-
**Beverages**[b]	54	38.9	19	45.2	35	36.1	0.309	26	52.0	27	32.9	**0.030**	39	41.1	12	33.3	0.419
Tea and coffee	17	12.2	5	11.9	12	12.4	0.939	5	10.0	12	14.6	0.441	10	10.5	5	13.9	0.589
Soft drinks and juices	10	7.2	3	7.1	7	7.2	0.988	4	8.0	6	7.3	0.886	8	8.4	2	5.6	0.581
Fermented traditional beverages[c]	7	5.0	3	7.1	4	4.1	0.455	5	10.0	2	2.4	0.060	5	5.3	2	5.6	0.947
Water	18	13.0	7	16.7	11	11.3	0.390	12	24.0	5	6.1	**0.003**	15	15.8	3	8.3	0.268
Alcoholic beverages[d]	2	1.4	1	2.4	1	1.0	0.539	0	0.0	2	2.4	0.266	1	1.1	0	0.0	0.537
Milk	2	1.4	0	0.0	2	2.1	0.349	0	0.0	2	2.4	0.266	2	2.1	0	0.0	0.380

Values in bold represent statistically significant differences according to Chi-squared test with a significance level of 0.05.

[a]For the variables age and weight status, the data presented corresponds to the customers in which there was agreement between observers (Tajikistan: n=67 and n=68; Kyrgyzstan: n=211 and n=215; Turkmenistan: n=232 and n=242; Kazakhstan: n=132 and n=131).

[b]The sum of the values for each sub-category may be higher than the total N of the respective category, because the same customer could buy more than one food item.

[c]Fermented traditional beverages included *ayran* (dairy-based fermented beverage made from sheep's milk), *chalap* (beverage made from fermented milk, salt and carbonated water;), *dugob* (fermented beverage made with sour milk or buttermilk), *kefir* (fermented milk drink prepared by inoculating cow, goat or sheep milk with kefir grains), *maksym* (fermented beverage made from grain, usually malt), *tamshan* (mix of *maksym* and *chalap*), k*ozhe* (cold drink made by boiling rice, millet or pearl barley with a mixture of dairy products such as *ayran* or *kefir*) and Yoghurt.

[d]Alcoholic beverages included beer, vodka and some traditional beverages with a low alcohol content, such as *kvass* (a fermented beverage made from rye bread), *bozo* (a fermented beverage made from millet) and *kymyz* (a fermented product made from mare's milk).
